# Two-year evolution of frailty status and predictive factors in Chinese older adults: a national longitudinal study

**DOI:** 10.1186/s12877-025-06541-0

**Published:** 2025-11-17

**Authors:** Jing Shi, Luyao Zhang, Yongkang Tao, Chao Gao, Yan Cen, Sainan Li, Ying Li, Botao Sang, Xiangfei Liu, Qinan Ma, Xuezai Zeng, Yan Zhang, Deping Liu

**Affiliations:** 1 The Key Laboratory of Geriatrics, Beijing Institute of Geriatrics, Institute of Geriatric Medicine, Chinese Academy of Medical Sciences, Beijing Hospital, National Center of Gerontology of National Health Commission, Beijing, 100730 China; 2 Department of Cardiology, Beijing Hospital, National Center of Gerontology, Institute of Geriatric Medicine, Chinese Academy of Medical Sciences, Beijing, 100730 China; 3 Peking Union Medical College, Graduate School of Peking Union Medical College, Chinese Academy of Medical Sciences, Beijing, 100730 China; 4 Department of Gastroenterology, China-Japan Friendship Hospital, Beijing, 100029 China; 5 Peking University Fifth School of Clinical Medicine, Beijing, 100730 China; 6 Savaid Medical School, University of Chinese Academy of Sciences, Beijing, 100730 China; 7 Department of General Practice/VIP Medical Service, Beijing Hospital, National Center of Gerontology, Institute of Geriatric Medicine, Chinese Academy of Medical Sciences, Beijing, 100730 China

**Keywords:** Frailty status, Predictive factors, Longitudinal study, National survey

## Abstract

**Background:**

With the global aging population, frailty in older adults has emerged as a critical focus in health and aging research. As a dynamic and multifactorial process, the transition to frailty is shaped by not only biological factors but also a range of social, psychological, and environmental influences. Identifying the key factors that drive the progression of frailty is essential for developing preventive interventions for at-risk individuals and for implementing more effective health practices and healthcare strategies for older adults.

**Methods:**

Data for this study were drawn from the Fourth Sample Survey of the Aged Population in Urban and Rural China, organized by the China National Committee on Ageing. The baseline data were collected from older individuals who participated in the 2017 survey, and the follow-up data were from the 2019 survey. Frailty in older adults was assessed using the Frailty Index (FI) model, which was used to examine the current frailty status among older adults in China and to prospectively analyze the developmental trajectory of frailty. Logistic regression was used to identify the factors influencing the progression of frailty.

**Results:**

A total of 9,093 older adults were included in the analysis. FI values increased with age and were consistently higher in women than in men, indicating that older women had higher levels of frailty. During the two-year follow-up period, frailty status remained stable in most older adults (56.2%, 5,111/9,093), while 1,292 (14.2%, 1,292/9,093) experienced an improvement and 2,690 (29.6%, 2,690/9,093) experienced a worsening of frailty. Transitions to a more frail state were more common than transitions to an improved state. Additionally, transitions between adjacent frailty statuses were much more frequent than transitions across multiple frailty categories (3,669 (40.3%) versus 313 (3.4%)). Logistic regression analysis identified several factors influencing the progression of frailty status in older adults, including demographic factors (age, sex, residence, education, and marital status); family and economic status (living alone, employment, pension receipt, home ownership, financial status); health and medical factors (exercise, recent illness, annual medical checkups, hospitalizations); caregiver support; and social participation (public welfare participation, involvement in senior associations, helping seniors in need, recreational participation, and regular internet access).

**Conclusion:**

The worsening of frailty with age is more common in older adults than the improvement of frailty. Among robust and pre-frail individuals, older women are more likely to experience a deterioration in frailty status. The factors influencing frailty transitions are multifaceted and complex. Therefore, when intervening in the progression of frailty among older adults, it is essential to comprehensively assess frailty risks and influencing factors based on the diverse characteristics and current status of individuals. Individualized, comprehensive, and targeted interventions and management strategies, tailored to different frailty stages and transition pathways, can help improve frailty in older adults.

## Background

Frailty has garnered significant attention as a prevalent syndrome among older adults. With the global aging, the negative consequences of frailty are becoming increasingly pronounced. Frailty is a complex geriatric condition characterized by a decline in physiological reserves and heightened vulnerability to stressors due to the dysregulation of multiple bodily systems. It affects between 4% and 59% of older adults, depending on the population and setting [1], and is strongly associated with adverse outcomes such as falls, fractures, hospitalization, disability, and mortality [2–4].

Importantly, frailty is not a static or irreversible condition. Unlike chronological aging, frailty is recognized as a dynamic state that can improve, worsen, or remain stable over time [5]. This dynamic nature highlights the potential for early identification and timely intervention. Previous studies have identified several factors associated with frailty progression, including advanced age, lower education level, lifestyle behaviors, and overall health status [6–8]. Moreover, social determinants of health—such as social participation, caregiver support, and economic circumstances—have been increasingly recognized as significant influences on frailty outcomes. For instance, a lower frequency of social interactions is linked to increased feelings of loneliness and depression [9]. Interactions with neighbors also provide essential social resources, including health information and emotional and practical support, which can act as important buffers against the worsening of frailty.

Despite these known associations, current researches in China have primarily focused on frailty screening and prevalence, with limited attention to the dynamic progression of frailty and its multidimensional influences in large population of community-dwelling older adults. Therefore, the aim of this study is to examine the progression of frailty and identify a wide range of factors—spanning demographic, economic, health, and social domains—that are associated with changes in frailty status over time. By identifying high-risk profiles within the general older adult population, the findings can help inform targeted health promotion strategies and guide resource allocation in community-based care. To achieve this, we conducted a prospective cohort study using data from the Fourth Sample Survey of the Aged Population in Urban and Rural China (SSAPUR), a nationally representative database. The results aim to support evidence-based approaches to frailty prevention and management in the aging Chinese population.

## Methods

### Study design and participants

Data for this study were sourced from the Fourth SSAPUR database, conducted by the China National Committee on Ageing beginning in 2015. The survey targeted Chinese citizens aged 60 years and older and covered 31 provinces, autonomous regions, and municipalities, resulting in the largest database of older adults in China. More detailed information on the survey design and sampling methods is available in previous studies [10, 11]. The study protocol was approved by the Ethics Review Committee of Beijing Hospital (No. 2021BJYYEC-294-01) and the National Bureau of Statistics (No. [2014] 87). All participants provided written informed consent.

The present cohort was a monitoring survey of the 2015 sample, with continuous tracking conducted in 2017 and 2019. Due to the impact of the pandemic, the 2017 and 2019 data remain the most recent available data on the aging population in China, and they form the largest sample of older individuals currently accessible for analysis. A total of 12,788 participants were included in the 2017 survey, which served as the baseline for this study. The 2019 survey database was used for follow-up. Frailty status was assessed in both 2017 and 2019, with frailty transition defined as the change in frailty status between these years.

Of the 12,788 older adults in 2017, 3,695 were lost to follow-up through 2019. Ultimately, 9,093 older adults were included in the analysis, with ages ranging from 62 to 101 years and a mean age of 71.2 ± 7.0 years. Among them, 4,598 (50.6%) were men and 4,495 (49.4%) were women. The majority of participants were Han Chinese (95.9%, 8,720 individuals). Urban older adults accounted for a slightly larger proportion than rural older adults [4,656 cases (51.2%) versus 4,437 cases (48.8%)].Most participants had a low level of education, with 6,633 cases (72.9%) being illiterate. Additionally, 72.6% (6,599 participants) had 1–3 comorbidities, and a vast majority (96.9%, 8,807 participants) had at least one activity of daily living (ADL) disability. At baseline in 2017, the mean frailty index (FI) for the 9,093 older adults was 0.14 ± 0.09. The numbers of robust, pre-frail, and frail individuals were 3,572 (39.3%), 4,098 (45.1%), and 1,423 (15.6%), respectively.

### Survey contents

The SSAPUR questionnaire was part of a large-scale epidemiological survey administered through face-to face interviews conducted by trained personnel. The survey encompassed nine key domains: basic demographic information, family structure, health status, care and nursing services, economic conditions, social participation, rights protection, livable environment, and spiritual and cultural life (including psychology well-being).

### Assessment of frailty and FI transitions

Frailty was assessed using the FI model developed by Professor Kenneth Rockwood’s team. The FI was constructed from 31 variables selected from the survey content, encompassing five categories: ADL (6 items), chronic diseases (11 items), geriatric syndromes (5 items), health status and emotional well-beiing (4 items), and use of assistive devices (5 items) [see Appendix 1]. Each variable was assigned a value based on its type—for example, dichotomous variables were coded as 0 or 1, while trichotomous variables were coded as 0, 0.5, or 1.These values ranged from 0 to 1, with higher scores indicating more severe health deficits [12].

The FI for each individual was calculated by dividing the total score of health deficits present by the total number of considered variables (31 in this study). The FI thus reflects the proportion of potential health deficits among all measured indicators. Like the assigned variable values, the FI ranges from 0 to 1, with higher scores denoting greater frailty and poorer health status [12].The FI served as the primary outcome variable in this study and was assessed at both baseline (2017) and follow-up (2019). Following the classification criteria established by Thompson et al. and Hoover et al. [13, 14], frailty status was categorized into three levels using FI cut-off points of 0.10 and 0.21: robust (FI ≤ 0.10), pre-frail (0.10 < FI ≤ 0.21), and frail (FI >0.21). Frailty transition (FI transition) over the two-year follow-up period (2017–2019) was defined as any change in frailty status, including worsening, stabilization, or improvement.

### Predictors

Transitions between frailty categories over the two-year period were analyzed to identify associated factors. As the primary aim of this study is to identify actionable risk profiles rather than to establish causal relationships, all variables were treated as predictors without categorizing them into covariates or primary independent variables. Based on the content of the survey questionnaire—and excluding variables used to construct the FI—the following predictors were considered: (1) Demographics: age group (60–, 70–, 80–), sex (men/women), ethnicity (minority/Han), residence (rural/urban), education (≥ Senior high school/≤Junior high school), and marital status (others including widowed, divorced or never married/married). (2) Family and economic status: living alone (yes/no), still in paid work (yes/no), receiving pension (yes/no), home ownership (yes/no), and self-reported financial status (poor/adequate/good). (3) Health and medical status: frequency of exercise per week (≥ 3 times/≤2 times), 2-week illness history (yes/no), annual medical checkups (yes/no), Hospitalizations in the past year (≥ 2 times/1 time/0), medicare coverage (yes/no), and ease of medical reimbursement (inconvenient/fair/convenient). (4) Caregiver support: availability of support during illness (yes/no). (5) Social participation: public welfare participation (yes/no), senior association participations (yes/no), helping seniors in need (yes/no), recreational participation (yes/no), regular internet access (yes/no), and online education participation (yes/no).

### Statistical analysis

Data analysis and visualization were conducted using SPSS version 24.0 and MATLAB 2020. Missing predictor values were addressed using multiple imputation via the Markov Chain Monte Carlo (MCMC) method [15]. Descriptive statistics were used to summarize the baseline characteristics of the study population. Continuous variables were presented as mean ± standard deviation (x ± s), while categorical variables were reported as percentages. To assess differences across frailty status groups, analysis of variance (ANOVA) was used for continuous variables, and chi-square tests were used for categorical variables. Histograms were constructed to illustrate the distribution of FI values. Bar charts were employed to compare FI scores between men and women across different age groups and to examine changes in frailty status among older adults with varying degrees of baseline frailty. Nonlinear regression models were applied to fit age-specific FI values as a function of age (modeled using an exponential function), stratified by sex at both baseline and follow-up.

To identify factors associated with frailty transitions, logistic regression models were used based on participants’baseline frailty status. Binary logistic regression was employed for participants who were either robust or frail at baseline (e.g., robust worsening vs. robust stability; frail improvement vs. frail stability). For those classified as pre-frail, multinomial logistic regression was used to model the three potential outcomes: worsening, improvement, and stability. Statistical significance was defined as a two-tailed *P*-value < 0.05 for all analyses.

## Results

### Comparison of general characteristics and associated factors among older adults with different frailty statuses

A total of 9,093 older adults were included in this study, with ages ranging from 62 to 101 years and a mean age of 71.2 ± 7.0 years. Among them, 4,495 were women (mean age: 71.5 ± 7.2 years) and 4,598 were men (mean age: 70.9 ± 6.8 years). The distribution of frailty status among participants was as follows: 39.3% were classified as robust (3,572/9,093), 45.1% as pre-frail (4,098/9,093), and 15.6% as frail (1,423/9,093). Higher proportions of pre-frailty and frailty were observed among older participants, women, individuals from ethnic minority groups, those residing in rural areas, and those with lower educational attainment.

The analysis was further stratified by gender. Among older women, frailty status varied significantly across different age groups, ethnicities, places of residence, and marital statuses. In older men, frailty status also exhibited significant variation according to age group, educational level, and marital status (all *P* < 0.05). Additionally, all other factors examined—except for annual medical checkups, medicare coverage, and participation in online education—were found to be associated with frailty status in both men and women (Table 1).

**Table 1:** Comparison of general characteristics and related factors among older adults with different levels of frailty

Variables		All (*n* = 9093)				Women (*n* = 4495)				Men (*n* = 4598)			
		Robust	Pre-frail	Frail	*P*-value	Robust	Pre-frail	Frail	*P*-value	Robust	Pre-frail	Frail	*P*-value
N		3572	4098	1423		1481	2157	857		2091	1941	566	
Demographics													
Age group	60-	48.5%	41.2%	10.3%	< 0.001	42.7%	44.5%	12.7%	< 0.001	53.8%	38.1%	8.1%	< 0.001
	70-	33.4%	49.0%	17.6%		26.9%	52.2%	20.9%		39.7%	45.8%	14.5%	
	80-	22.8%	48.6%	28.7%		17.1%	49.0%	33.9%		29.4%	48.1%	22.5%	
Sex	Women	32.9%	48.0%	19.1%	< 0.001								
	Men	45.5%	42.2%	12.3%									
Ethnicity	Han	39.6%	44.8%	15.6%	0.034	33.3%	47.6%	19.2%	0.017	45.7%	42.1%	12.2%	0.114
	Minority	33.0%	50.7%	16.4%		24.9%	58.6%	16.6%		39.7%	44.1%	16.2%	
Residence	Urban	41.2%	44.1%	14.7%	< 0.001	35.5%	46.9%	17.7%	< 0.001	47.1%	41.2%	11.7%	0.071
	Rural	37.3%	46.1%	16.6%		30.1%	49.2%	20.6%		43.9%	43.2%	12.9%	
Education	≤Junior high school	32.5%	51.8%	15.7%	0.024	32.9%	48.0%	19.1%	0.591	45.9%	41.8%	12.3%	0.009
	≥Senior high school	43.7%	45.2%	11.1%		66.7%	33.3%	0.0%		33.3%	55.3%	11.4%	
Marital status	Married	43.3%	43.3%	13.4%	< 0.001	37.7%	46.5%	15.7%	< 0.001	47.6%	40.9%	11.6%	< 0.001
	Others^a^	28.2%	49.9%	22.0%		24.4%	50.6%	25.0%		35.9%	48.4%	15.8%	
Family and economic status													
Living alone		28.9%	50.8%	20.3%	< 0.001	23.7%	52.6%	23.8%	< 0.001	36.1%	48.4%	15.5%	< 0.001
Still in paid employment		64.2%	32.2%	3.6%	< 0.001	55.1%	38.8%	6.1%	< 0.001	67.9%	29.5%	2.6%	< 0.001
Receiving pension		49.9%	40.8%	9.2%	< 0.001	43.3%	44.7%	12.0%	< 0.001	55.6%	37.5%	6.9%	< 0.001
Home ownership		42.5%	44.2%	13.3%	< 0.001	36.7%	46.9%	16.4%	< 0.001	47.4%	41.9%	10.7%	< 0.001
Financial status	Good	58.4%	35.3%	6.3%	< 0.001	51.6%	40.8%	7.6%	< 0.001	64.1%	30.6%	5.2%	< 0.001
	Adequate	41.1%	45.9%	13.0%		34.3%	49.3%	16.4%		48.0%	42.4%	9.6%	
	Poor	17.1%	51.5%	31.4%		13.9%	50.3%	35.8%		20.4%	52.7%	26.9%	
Health and medical status													
Exercise/week	≤ 2 times	36.6%	45.3%	18.1%	< 0.001	30.9%	47.7%	21.5%	< 0.001	42.7%	42.8%	14.5%	< 0.001
	≥ 3 times	43.0%	44.8%	12.2%		36.2%	48.5%	15.3%		48.8%	41.5%	9.6%	
2-week illness history		15.5%	49.8%	34.8%	< 0.001	13.7%	48.7%	37.7%	< 0.001	18.0%	51.4%	30.6%	< 0.001
Annual medical checkups		38.7%	45.7%	15.6%	0.150	33.3%	48.1%	18.6%	0.400	44.1%	43.3%	12.6%	0.027
Hospitalizations (past year)	0	45.0%	42.7%	12.4%	< 0.001	38.1%	46.3%	15.6%	< 0.001	51.5%	39.2%	9.3%	< 0.001
	1 time	24.9%	54.2%	21.0%		20.7%	56.2%	23.2%		28.9%	52.3%	18.8%	
	≥ 2 times	13.2%	49.4%	37.3%		9.0%	48.3%	42.6%		18.1%	50.8%	31.1%	
Medicare coverage		39.3%	45.1%	15.6%	0.667	33.0%	48.0%	19.0%	0.499	45.5%	42.2%	12.3%	0.924
Medical reimbursement	Convenient	39.8%	45.0%	15.2%	< 0.001	33.2%	48.2%	18.7%	< 0.001	46.2%	42.0%	11.8%	< 0.001
	Fair	31.9%	49.5%	18.6%		27.2%	52.4%	20.4%		36.8%	46.5%	16.7%	
	inconvenient	24.1%	49.4%	26.6%		18.2%	51.3%	30.5%		29.6%	47.5%	22.8%	
Caregiver support													
Support during illness		3.2%	31.8%	64.9%	< 0.001	2.9%	30.6%	66.5%	< 0.001	3.7%	33.5%	62.8%	< 0.001
Social participation													
Public welfare participation		43.9%	44.3%	11.9%	< 0.001	37.2%	47.6%	15.1%	< 0.001	49.6%	41.4%	9.0%	< 0.001
Senior associations participation		47.1%	41.9%	11.0%	< 0.001	38.8%	46.6%	14.6%	< 0.001	54.6%	37.6%	7.8%	< 0.001
Helping seniors in need		41.5%	44.5%	14.0%	< 0.001	34.8%	47.8%	17.3%	< 0.001	47.8%	41.3%	10.8%	< 0.001
Recreational participation		40.4%	45.1%	14.5%	< 0.001	34.4%	48.2%	17.4%	< 0.001	46.0%	42.3%	11.8%	< 0.001
Regular internet access		53.8%	40.0%	6.2%	< 0.001	45.1%	48.6%	6.3%	< 0.001	59.0%	34.9%	6.1%	< 0.001
Online education participation		47.6%	43.5%	8.9%	0.003	44.1%	47.5%	8.5%	0.003	50.8%	39.8%	9.4%	0.391

^a^Other marital status includes widowed, divorced or never married

### Analysis of frailty status in older adults at baseline and follow-up

**Fig. 1:**
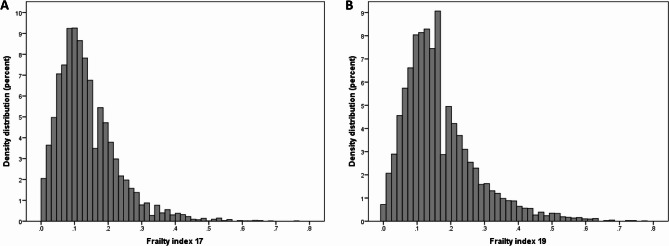
Distributions of frailty index (FI): panel (**A**) older adults at baseline in 2017.Panel (**B**) older adults at follow-up in 2019

**Fig. 2:**
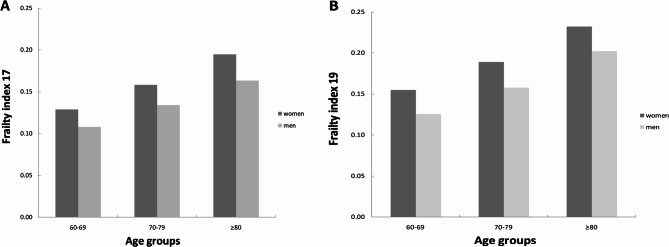
Mean frailty index in relation to age and sex. Panel (**A**) older adults at baseline in 2017. Panel (**B**) older adults at follow-up in 2019

At baseline in 2017, the 9,093 older adults had a minimum FI value of 0, a maximum value of 0.76, a median of 0.12, a mode of 0.09, and a mean value of 0.14 ± 0.09. Specifically, men had a median FI value of 0.10 and a mean of 0.12 ± 0.08, while women had a median FI value of 0.14 and a mean of 0.15 ± 0.09. At the 2019 follow-up, the minimum FI value was 0, the maximum value was 0.77, the median was 0.14, and the mean FI value was 0.16 ± 0.11. For men, the median FI value was 0.13, with a mean of 0.15 ± 0.10, whereas women had a median FI value of 0.15 and a mean of 0.18 ± 0.11 (Fig. 1A–B). FI values were compared between men and women across different age groups. FI values increased with age, and women were found to be more frail than men in all age groups (Fig. 2A–B).

**Fig. 3:**
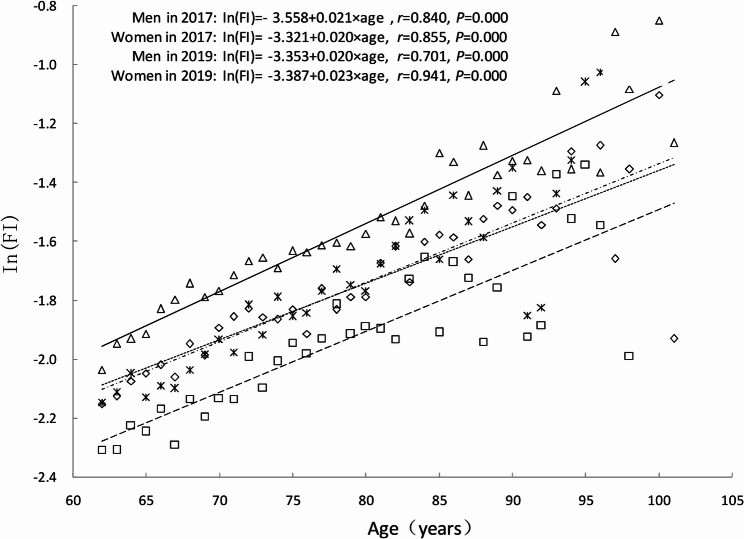
The relationship between age and the mean value of frailty index (FI) at baseline in 2017 and at follow-up in 2019. Men in 2017: square and dashed line; Women in 2017: diamond and dotted line; Men in 2019: star and dashed and dotted line; Women in 2019: triangle and solid line

Subsequently, trends in FI values across age groups were analyzed for older adults of different sexes. FI values increased exponentially with age in both older men and women, as represented by the model Ln(FI) = A + BXage. Women consistently exhibited higher FI values than men at all ages, both at baseline in 2017 and at follow-up in 2019. Notably, the frailty status of older men at the 2019 follow-up was comparable to that of older women at baseline in 2017 (Fig. 3). On a logarithmic scale, at baseline in 2017, older women showed a slightly lower rate of health deficit accumulation compared to men; however, this differences was statistically insignificant (B = 0.020 vs. B = 0.021, *t* = 1.049, *P* = 0.639). In contrast, by 2019, the mean annual relative growth rate of FI values was higher in women than in men (B = 0.023 vs. B = 0.020, *t* = 5.100, *P* < 0.001). This indicates that older women accumulated health deficits at a faster rate than men (Fig. 3).

### Changes in frailty status

**Fig. 4:**
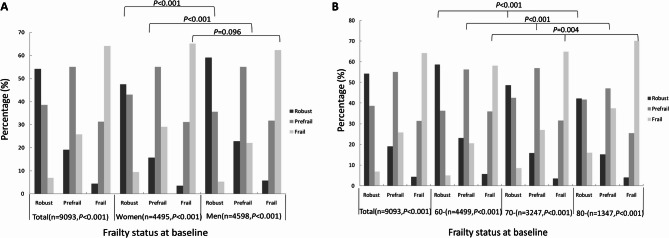
Changes in frailty status from baseline to follow-up: (**A**) according to sex, (**B**) according to age group

At baseline in 2017, the proportions of robust, pre-frail, and frail older adults were 39.3% (3,572/9,093), 45.1% (4,098/9,093), and 15.6% (1,423/9,093), respectively.By 2019, these proportions changed to 30.6% (2,784/9,093), 45.0% (4,089/9,093), and 24.4% (2,220/9,093). Over the 2-year follow-up, the frailty status of most older adults remained stable (56.2%, 5,111/9,093). However, 14.2% (1,292/9,093) experienced an improvement in frailty, while 29.6% (2,690/9,093) showed worsening frailty. Among those who remained in the same frailty category, 54.3% (1,939/3,572) of robust older adults, 55.1% (2,259/4,098) of pre-frail older adults, and 64.2% (913/1,423) of frail older adults did not change from their baseline frailty status. In terms of frailty transitions, 38.7% (1,383/3,572) of baseline robust older adults transitioned to pre-frail status, 25.8% (1,057/4,098) of pre-frail older adults progressed to frailty, and 7.0% (250/3,572) of robust older adults directly progressed to frailty. Notably, 31.4% (447/1,423) of frail older adults improved to a pre-frail status at the 2-year follow-up, 19.1% (782/4,098) of pre-frail older adults improved to a robust status, and 4.4% (63/1,423) of frail older adults reverted to robust status. Overall, transitions to more frail statuses (i.e., deterioration) were more common than improvements in frailty. Furthermore, transitions between adjacent frailty statuses occurred more frequently than transitions spanning multiple frailty stages [3,669 (40.3%) vs. 313 (3.4%)] (Fig. 4).

Further sex subgroup analyses revealed that among older women, 54.6% (2,453/4,495) had stable frailty statuses, 14.1% (635/4,495) showed improvement, and 31.3% (1,407/4,495) experienced worsening frailty. In contrast, among older men, 57.8% (2,658/4,598) had stable frailty statuses, 14.3% (657/4,598) showed improvement, and 27.9% (1,283/4,598) experienced worsening frailty. Overall, a higher proportion of women than men experienced worsening frailty (χ^2^ = 13.148, *P* = 0.001). Specifically, among robust and pre-frail older adults, a higher proportion of men than women maintained stable or improved frailty statuses, while women were more likely to experience worsening frailty (χ^2^ = 55.849, 47.820, both *P* < 0.001). However, among frail older adults, the difference in frailty status transitions between men and women was not statistically significant (χ^2^ = 4.695, *P* = 0.096) (Table 2; Fig. 4A). Age subgroup analyses showed that in the 60 + group, 57.7% (2,595/4,499) of older adults had stable frailty statuses, 13.8% (623/4,499) showed improvement, and 28.5% (1,281/4,499) experienced worsening frailty. In the 70 + group, these proportions were 55.6% (1,806/3,247), 14.0% (454/3,247), and 30.4% (987/3,247), respectively. For the 80 + group, the proportions were 52.7% (710/1,347), 16.0% (215/1,347), and 31.3% (422/1,347) (Table 2; Fig. 4B). Notably, the risk of worsening frailty increased with age (χ^2^ = 12.297, *P* = 0.015). Additionally, the association between age and frailty transition was significant across all baseline frailty statuses (robust, pre-frail, or frail) (χ^2^ = 82.809, 93.467, 15.251, all *P* < 0.05). In other words, older adults were more likely to experience worsening frailty as age increased (Table 2; Fig. 4B).

**Table 2:** Changes in frailty status among the study participants according to sex and age

Characteristics	Robust to			Pre-frail to			Frail to			Total to		
	Robust (*n* = 1939)	Pre-frail (*n* = 1383)	Frail (*n* = 250)	Robust (*n* = 782)	Pre-frail (*n* = 2259)	Frail (*n* = 1057)	Robust (*n* = 63)	Pre-frail (*n* = 447)	Frail (*n* = 913)	Robust (*n* = 2784)	Pre-frail (*n* = 4089)	Frail (*n* = 2220)
Gender												
Women	703(47.5)	638(43.1)	140(9.5)	338(15.7)	1190(55.2)	629(29.2)	30(3.5)	267(31.2)	560(65.3)	1071(23.8)	2095(46.6)	1329(29.6)
Men	1236(59.1)	745(35.6)	110(5.3)	444(22.9)	1069(55.1)	428(22.1)	33(5.8)	180(31.8)	353(62.4)	1713(37.3)	1994(43.4)	891(19.4)
X^2^	55.849			47.820			4.695			235.822		
*P*-value	< 0.001			< 0.001			0.096			< 0.001		
Agegroup												
60-	1281(58.7)	792(36.3)	108(5.0)	429(23.1)	1044(56.3)	381(20.6)	27(5.8)	167(36.0)	270(58.2)	1737(38.6)	2003(44.5)	759(16.9)
70-	528(48.7)	463(42.7)	93(8.6)	253(15.9)	906(57.0)	431(27.1)	20(3.5)	181(31.6)	372(64.9)	801(24.7)	1550(47.7)	896(27.6)
80-	130(42.3)	128(41.7)	49(16.0)	100(15.3)	309(47.2)	245(37.5)	16(4.1)	99(25.6)	271(70.2)	246(18.3)	536(39.8)	565(41.9)
X^2^	82.809			93.467			15.251			500.159		
*P*-value	< 0.001			< 0.001			0.004			< 0.001		

### Logistic regression analysis of factors influencing frailty transition in older adults

The baseline FI values for older adults whose frailty status remained stable, worsened, or improved were 0.14 ± 0.10, 0.10 ± 0.05, and 0.19 ± 0.07, respectively. Individuals with higher baseline frailty were more likely to experience improvements in their frailty statuses (*F* = 423.633, *P* < 0.001). Logistic regression analyses were conducted to investigate the factors influencing frailty transitions at different baseline frailty levels. The results are as follows: (1) Demographics: Among older adults with different frailty statuses, increasing age, being female, and living in a rural area were associated with worsened frailty status. However, being widowed, divorced, or unmarried, as well as having a low level of education, increased the risk of worsened frailty status only among robust and pre-frail older adults; these factors did not correlate with improved frailty status in frail older adults. Additionally, ethnic minority older adults were more likely to experience frailty improvement compared to Han Chinese older adults. (2) Family and economic status: Living alone increased the risk of worsened frailty status among pre-frail older adults. However, for frail older adults, living alone was associated with a higher likelihood of frailty improvement. Among robust and pre-frail older adults, being in paid work, receiving a pension, owning a home, and having good financial status reduced the risk of worsened frailty, but these factors had no effect on frail older adults. (3) Health and medical status: Engaging in more weekly exercise decreased the risk of worsened frailty but had no effect on frail older adults. Additionally, a 2-week illness history increased the risk of worsened frailty among robust older adults. Annual medical checkups reduced the risk of worsened frailty in robust older adults, but had no effect on pre-frail and frail older adults. Hospitalizations in the past year increased the risk of worsened frailty for both robust and pre-frail older adults. (4) Caregiver support: Providing support during illness had a positive effect on frailty improvement among pre-frail and frail older adults.(5) Social participation: Regular engagement in public welfare, senior associations, helping seniors in need, recreational activities (such as playing ball games or fishing), and internet access decreased the risk of worsened frailty, especially for robust or pre-frail older adults (Table 3).

**Table 3:** Logistic regression for factors influencing 2-year changes in frailty among the study participants [*OR*(95%*CI*)]

Variables	Total				Women				Men			
	Robust worsening	Pre-frail worsening	Pre-frail improvement	Frail improvement	Robust worsening	Pre-frail worsening	Pre-frail improvement	Frail improvement	Robust worsening	Pre-frail worsening	Pre-frail improvement	Frail improvement
Demographics												
Age group	1.378* (1.167,1.533)	1.362* (1.219,1.637)	0.913* (0.524,0.973)	0.858* (0.328,0.921)	1.312* (1.119,1.512)	1.415* (1.203,1.551)	0.772 (0.591,1.118)	0.767* (0.552,0.989)	1.610* (1.259,1.736)	1.615* (1.299,2.010)	0.799* (0.573,0.976)	0.667* (0.512,0.903)
Sex	0.439* (0.216,0.805)	0.769* (0.466,0.911)	1.533* (1.241,1.723)	1.310* (1.132,1.419)								
Ethnicity	0.696 (0.428,1.377)	0.765 (0.579,1.519)	1.179 (0.589,1.690)	2.339* (1.514,4.079)	0.693 (0.422,1.439)	1.119 (0.594,1.579)	1.427 (0.766,2.593)	1.372 (0.598,2.991)	0.989 (0.582,1.671)	0.799 (0.454,1.511)	0.811 (0.496,1.317)	2.579* (1.362,4.779)
Residence	1.302* (1.053,1.404)	1.038 (0.677,1.591)	0.638* (0.413,0.905)	0.557* (0.482,0.897)	1.312* (1.109,1.669)	1.092 (0.778,1.342)	0.783 (0.656,1.231)	0.695* (0.412,0.875)	1.310* (1.059,1.714)	1.113 (0.724,1.344)	0.557* (0.469,0.901)	0.561* (0.311,0.886)
Education	0.747* (0.437,0.962)	0.711* (0.529,0.960)	1.211* (1.017,1.526)	1.317 (0.799,1.510)	0.742 (0.553,1.397)	0.669* (0.562,0.916)	1.415* (1.139,2.512)	1.313 (0.765,1.831)	0.811 (0.593,1.297)	0.869 (0.665,1.378)	1.103 (0.811,1.342)	1.179 (0.711,1.896)
Marital status	1.423* (1.115,1.986)	1.378* (1.215,1.794)	0.699* (0.435,0.978)	0.910 (0.542,1.117)	1.216 (0.887,1.497)	1.349* (1.117,1.654)	0.667* (0.417,0.899)	0.801 (0.492,1.113)	1.277* (1.113,1.704)	1.210 (0.715,1.926)	0.891 (0.659,1.321)	1.117 (0.669,1.395)
Family and economic status												
Living alone	1.213 (0.966,1.529)	1.372* (1.102,1.591)	0.573* (0.442,0.791)	1.342* (1.121,1.834)	1.159 (0.769,1.543)	1.351* (1.119,1.735)	0.679* (0.358,0.912)	1.311 (0.889,1.661)	1.197 (0.876,1.566)	1.330 (0.835,1.715)	0.803 (0.515,1.225)	1.642* (1.129,3.156)
Still in paid employment	0.529* (0.396,0.711)	0.465* (0.319,0.576)	1.616* (1.139,2.223)	1.323 (0.697,2.838)	0.615* (0.433,0.900)	0.495* (0.312,0.911)	0.869 (0.443,1.502)	1.159 (0.476,3.211)	0.597* (0.488,0.943)	0.491* (0.287,0.801)	1.735* (1.266,2.433)	2.010 (0.751,5.110)
Receiving pension	0.726* (0.589,0.933)	0.669* (0.543,0.923)	1.034 (0.766,1.290)	1.113 (0.546,1.378)	0.711* (0.532,0.895)	0.558* (0.433,0.892)	1.023 (0.715,1.348)	0.903 (0.579,1.366)	0.706 (0.593,1.117)	0.715 (0.654,1.169)	0.869 (0.657,1.335)	0.842 (0.554,1.391)
Home ownership	0.804* (0.635,0.977)	0.780* (0.660,0.935)	1.015 (0.794,1.238)	1.210 (0.866,1.436)	0.898 (0.656,1.223)	0.816* (0.625,0.992)	1.110 (0.745,1.510)	1.119 (0.796,1.521)	0.743* (0.409,0.954)	0.711 (0.594,1.115)	0.822 (0.616,1.117)	1.178 (0.831,1.597)
Financial status	1.312* (1.171,1.563)	1.295* (1.113,1.464)	1.010 (0.866,1.217)	0.997 (0.693,1.324)	1.310* (1.114,1.692)	1.326* (1.135,1.607)	0.885 (0.767,1.210)	0.824 (0.512,1.259)	1.245* (1.096,1.498)	1.121 (0.858,1.422)	1.103 (0.859,1.251)	0.868 (0.623,1.112)
Health and medical status												
Exercise/week	0.836* (0.771,0.982)	0.764* (0.463,0.859)	1.054 (0.882,1.174)	1.135 (0.768,1.279)	0.915 (0.775,1.054)	0.922* (0.734,0.998)	1.115 (0.856,1.231)	1.033 (0.898,1.156)	0.879* (0.766,0.993)	0.865 (0.742,1.116)	1.106* (1.011,1.314)	1.120 (0.896,1.295)
2-week illness history	1.435* (1.117,3.719.)	1.233 (0.665,2.194)	1.017 (0.733,1.219)	0.874 (0.591,2.319)	1.270* (1.110,1.656)	1.115 (0.812,1.544)	0.596 (0.326,1.695)	0.913 (0.688,1.333)	1.381 (0.952,1.996)	1.209 (0.884,1.657)	0.912 (0.677,1.310)	0.815 (0.549,1.216)
Annual medical checkups	0.679* (0.483,0.889)	0.876 (0.713,1.392)	1.031 (0.689,1.135)	1.027 (0.776,1.258)	0.569* (0.483,0.812)	0.877 (0.591,1.230)	1.112 (0.836,1.185)	1.136 (0.769,1.402)	0.755* (0.579,0.902)	0.969 (0.774,1.328)	0.916 (0.720,1.332)	0.885 (0.556,1.318)
Hospitalizations (past year)	1.259* (1.106,1.796)	1.603* (1.315,2.149)	0.609* (0.495,0.931)	0.579 (0.362,1.497)	1.379* (1.124,1.779)	1.233* (1.131,1.526)	0.661* (0.479,0.913)	0.842 (0.684,1.112)	1.543* (1.226,2.005)	1.279* (1.078,1.504)	0.877 (0.655,1.312)	0.896 (0.662,1.118)
Medicare coverage	0.654 (0.363,1.896)	0.735 (0.346,1.538)	1.279 (0.433,3.665)	1.311 (0.393,3.259)	0.689 (0.157,2.596)	0.778 (0.274,2.259)	1.182 (0.369,4.467)	1.115 (0.378,4.934)	0.612 (0.334,1.675)	0.686 (0.235,2.501)	3.050 (0.403,22.967)	2.052 (0.311,17.392)
Medical reimbursement	1.017 (0.896,1.172)	0.766 (0.549,1.138)	1.117 (0.815,1.396)	0.962 (0.782,1.295)	1.110 (0.815,1.359)	1.005 (0.716,1.238)	1.059 (0.573,1.311)	0.859 (0.366,1.329)	1.113 (0.921,1.236)	0.995 (0.874,1.210)	0.861 (0.744,1.215)	0.896 (0.711,1.115)
Caregiver support												
Support during illness	0.862 (0.479,2.157)	0.671 (0.365,1.215)	1.687* (1.196,3.556)	2.359* (1.336,2.988)	0.591 (0.212,3.556)	0.835 (0.479,1.632)	1.882 (0.673,4.211)	2.859* (1.862,5.330)	0.655 (0.179,2.667)	0.339 (0.156,1.992)	2.331* (1.149,5.316)	2.852* (1.091,3.667)
Social participation												
Public welfare participation	0.759 (0.768,1.005)	0.633* (0.379,0.856)	1.257* (1.169,2.101)	0.976 (0.755,1.241)	0.953 (0.537,1.079)	0.779* (0.541,0.864)	0.887 (0.654,1.326)	1.152 (0.764,1.616)	0.951 (0.808,1.214)	0.785* (0.498,0.916)	1.705* (1.311,2.456)	1.201 (0.947,1.505)
Senior associations participation	0.897 (0.669,1.125)	0.792* (0.571,0.966)	1.066 (0.799,1.321)	1.052 (0.659,1.477)	0.879 (0.656,1.211)	0.711* (0.450,0.927)	1.121 (0.811,1.537)	1.115 (0.701,1.622)	0.843 (0.651,1.169)	0.865 (0.554,1.371)	1.310 (0.886,1.716)	1.113 (0.556,1.781)
Helping seniors in need	0.637* (0.469,0.857)	0.756* (0.362,0.882)	1.002 (0.815,1.177)	1.291 (0.986,1.759)	0.775 (0.467,1.176)	0.668* (0.436,0.891)	0.815 (0.597,1.231)	1.137 (0.866,1.710)	0.755* (0.543,0.956)	0.879 (0.569,1.347)	1.115 (0.764,1.510)	1.407 (0.896,2.137)
Recreational participation	0.853 (0.596,1.235)	0.722 (0.433,1.124)	1.338* (1.112,2.359)	1.411 (0.869,1.846)	0.793 (0.466,1.310)	0.808 (0.569,1.216)	1.803* (1.115,3.251)	1.324 (0.769,2.015)	0.761 (0.482,1.638)	0.979 (0.467,1.835)	0.831 (0.537,1.768)	1.497 (0.663,3.164)
Regular internet access	0.703* (0.549,0.866)	0.689 (0.412,1.215)	1.223 (0.796,1.591)	1.672 (0.819,3.739)	0.710* (0.456,0.976)	1.117 (0.654,1.704)	1.843* (1.163,3.579)	1.565 (0.606,4.237)	0.810 (0.459,1.113)	0.557* (0.309,0.872)	1.224 (0.756,1.927)	2.003 (0.873,4.517)
Online education participation	0.667 (0.437,1.159)	0.671 (0.441,1.068)	1.297 (0.776,2.392)	1.259 (0.477,3.014)	0.556 (0.325,1.211)	0.651 (0.352,1.315)	1.304 (0.612,3.115)	1.389 (0.321,4.694)	0.804 (0.442,1.582)	0.469 (0.233,1.227)	1.459 (0.662,3.015)	1.284 (0.477,3.719)

*indicates statistical significance

Further subgroup analysis by sex revealed the impact of various factors on frailty transition: (1) Demographics: age and place of residence affected frailty transitions in both older men and women. However, frail ethnic minority men were more likely to improve their frailty status compared to Han Chinese frail men. Educational attainment influenced frailty transitions only among pre-frail women. Additionally, marital status primarily affected pre-frail older women and robust older men. (2) Family and economic status: Living alone influenced frailty transitions in pre-frail older women and frail older men. All economic status variables were associated with frailty transitions in both men and women, except for receiving a pension, which only affected older women. (3) Health and medical status: The frequency of weekly exercise and hospitalizations in the past year influenced frailty transitions in both older men and women.A 2-week illness history affected only robust women. Moreover, annual medical checkups provided protection against frailty transitions in robust individuals but had no effect on pre-frail or frail individuals. (4) Caregiver support: Caregiver support during illness positively influenced frailty improvement, especially in frail older women and pre-frail and frail older men.(5) Social participation: With the exception of participation in online education, social participation positively affected frailty transitions among robust or pre-frail older men and women but had minimal impact on frail individuals (Table 3).

## Discussion

Using data from the SSAPUR database, this prospective cohort study examined frailty status and its natural transitions among community-dwelling older adults in China. The study also identified key factors influencing changes in frailty over time. Additionally, the frailty status of older adults was evaluated using the FI model. Frailty was assessed using the FI, a tool widely regarded as the most comprehensive method for evaluating frailty, as it encompasses a broad range of associated factors. The FI is considered especially valuable in both routine clinical care and community settings [16]. The FI conceptualizes frailty as a continuum of health, making it particularly suitable for capturing the dynamic nature of frailty in the general older population [17].

This study found that FI scores increased with age at both baseline (2017) and follow-up (2019). Additionally, at all ages, older women were more likely to be frail than their male counterparts, consistent with prior findings [18, 19]. Biological, psychological, and social differences between men and women may contribute to this disparity. Older women are more prone to social isolation, emotional stress, and negative psychological states, all of which can elevate frailty risk [20, 21]. Hormonal changes also play a crucial role in frailty development. Declines in sex hormone levels are associated with muscle weakness, fatigue, and functional decline. After menopause, women experience a sharp drop in estrogen, increasing their vulnerability to physical symptoms (e.g., fatigue, insomnia, headache) and psychological conditions (e.g., anxiety, depression) [22]. In contrast, frailty in older men is more closely linked to androgen levels, and higher levels of serum-free testosterone and dihydrotestosterone are associated with a lower risk of frailty [23]. These findings highlight the importance of sex-specific approaches in frailty intervention. Tailoring strategies based on the unique physiological and psychosocial characteristics of older men and women may enhance the effectiveness of frailty prevention and management.

Frailty is a dynamic and potentially reversible condition. Over the 2-year follow-up period of this study, the majority of older adults (56.2%) maintained a stable frailty status, 14.2% showed improvement, and 29.6% experienced worsening frailty. Notably, only 4.4% of frail individuals returned to a robust (healthy) state. These findings indicate that deterioration in frailty status was more common than improvement, and that transitions between adjacent frailty levels occurred more frequently than transitions across multiple levels. These results are consistent with findings from previous studies. For example, the Survey of Health, Ageing and Retirement in Europe (SHARE), which included 14,424 adults aged 55 and older across 11 European countries, reported that over a 2-year period, 61.8% had unchanged frailty, 22.1% experienced worsening, and 16.1% showed improvement [24]. Similarly, a U.S. study found that frailty deterioration was more prevalent than improvement, with recovery from frailty to a robust state occurring in only 0–0.9% of individuals over 18-month follow-up intervals [25]. These findings highlight the importance of early interventions, as preventing the onset or progression of frailty may be more achievable than reversing it once fully developed.

Subgroup analysis by sex in the present study revealed that women were more likely than men to experience worsening frailty, particularly among those initially robust or pre-frail. However, no statistically significant sex difference was observed in frailty transitions among individuals who were already frail. This aligns with the findings of Lorenzo-López et al. [26] and Ye et al. [27]. These observations underscore the need to consider sex-related disparities in physical and mental health during the early stages of frailty. Women may be more vulnerable to factors that accelerate frailty progression. However, once frailty is established, other health-related and social determinants—rather than sex alone—may play a more decisive role in influencing further decline.

In the present study, logistic regression analysis identified several key factors influencing the transition to frailty among older adults. Among demographic characteristics, increasing age, female sex, and residence in rural areas were significantly associated with worsening frailty status—findings consistent with previous research. These results underscore the importance of social and geographic determinants in the frailty trajectory. Specifically, older adults living in rural areas face heightened frailty and health risks, likely due to limited access to medical and healthcare resources [28]. Furthermore, being widowed, divorced, or unmarried, as well as having a low level of educational attainment, was associated with a higher risk of frailty progression in robust and pre-frail individuals. However, these factors appeared to have limited impact among those already classified as frail, suggesting that biological and physiological factors may play a more prominent role in later stages of frailty development [29]. Notably, frail older adults from ethnic minority groups showed a higher likelihood of frailty improvement compared to their Han Chinese counterparts, aligning with previous studies that highlight the beneficial effects of strong community and cultural support on frailty outcomes [30]. These findings suggest that specific social and cultural contexts may offer older adults greater psychological and social support, thereby facilitating improvements in frailty status and overall health. Living alone was found to significantly increase the risk of frailty progression in pre-frail individuals, reinforcing the importance of social support in preventing the worsening of frailty, as previously reported [31]. Interestingly, for those already frail, living alone appeared to be associated with frailty improvement, potentially reflecting adaptive coping mechanisms and enhanced self-management among these individuals. Nonetheless, further research is warranted to better understand this complex dynamic. In addition, robust and pre-frail older adults who were still engaged in paid employment, received pensions, owned homes, and reported good financial status experienced a significantly lower risk of frailty progression—findings that are consistent with prior literature [30]. These results highlight the protective role of financial stability and asset ownership in mitigating frailty risk. However, these socioeconomic factors appeared to exert minimal influence among those who were already frail, suggesting that the protective effects of economic resources may diminish as health significantly deteriorates. Medical conditions, such as frequent hospitalizations and recent illnesses, were also found to elevate the risk of frailty worsening, in line with previous findings [32]. Importantly, these adverse health factors had a more pronounced effect on robust and pre-frail individuals, emphasizing the critical need for early interventions targeting at-risk populations. The study also revealed that receiving support during illness had a beneficial effect on frailty improvement among pre-frail and frail individuals, underscoring the vital role of social and emotional support in promoting health and well-being [33]. Moreover, findings related to social participation emphasized the protective effect of active engagement in social and leisure activities against frailty progression [34]. Lastly, the study identified sex-specific differences in responses to frailty-related risk factors and highlighted the varying needs for prevention and intervention strategies between older men and women. Accordingly, a sex-stratified analysis was conducted to better understand these distinctions. Overall, the findings provide valuable insights into the multifactorial influences on frailty transitions in older adults and support the development of targeted, individualized intervention strategies tailored to sex and frailty status.

This study has several limitations. First, the reliance on self-reported data may introduce information bias, particularly given that older adults with cognitive impairments may inaccurately report their health status and lifestyle behaviors. Second, although this study examined differences in the factors influencing frailty transitions between male and female older adults, it did not explore the potential interactions between sex and other social or economic variables in depth. Additionally, the selection of variables was limited to those included in the SSAPUR questionnaire, potentially omitting important confounders such as genetic predispositions and long-term lifestyle factors, including smoking and alcohol consumption, which are known to affect the onset and progression of frailty. Future research should consider refining the questionnaire to incorporate these additional factors, enabling more comprehensive and nuanced analyses.

## Conclusions

In conclusion, this study utilized data from the SSAPUR database to investigate the transition to frailty and its associated factors among older adults. The data’s size and scope make it highly representative, providing valuable insights into the current state of aging in China. The findings indicate that frailty status generally tends to deteriorate with age rather than improve. Notably, among robust and pre-frail individuals, women were more likely to experience worsening frailty status. The progression of frailty is shaped by a complex interplay of factors, including age, sex, place of residence, participation in social activities, and economic status. Given this multifactorial nature, interventions aimed at preventing or mitigating frailty should take into account the diverse characteristics and current conditions of older adults. Comprehensive assessments of frailty risk and contributing factors, combined with stage-specific and personalized intervention strategies, are essential for effectively addressing frailty and promoting healthy aging.

## Data Availability

The data may be available from the corresponding author on reasonable request.

## Appendix 1

**Table 4:** Variables and calculation of frailty index

Frailty index	Cut-off
ADL	
1. Feeding	Yes = 0, with difficulty = 0.5, need help = 1
2. Getting dressed	Yes = 0, with difficulty = 0.5, need help = 1
3. Using the toilet	Yes = 0, with difficulty = 0.5, need help = 1
4. Getting in and out of bed	Yes = 0, with difficulty = 0.5, need help = 1
5. Walking indoors	Yes = 0, with difficulty = 0.5, need help = 1
6. Bathing	Yes = 0, with difficulty = 0.5, need help = 1
Chronic diseases	
7. Glaucoma/cataracts	Yes = 1, no = 0
8. Hypertension	Yes = 1, no = 0
9. Diabetes	Yes = 1, no = 0
10.Cardiovascular and cerebrovascular diseases	Yes = 1, no = 0
11.Gastric diseases	Yes = 1, no = 0
12. Osteoarthritis	Yes = 1, no = 0
13. Chronic lung disease	Yes = 1, no = 0
14.Asthma	Yes = 1, no = 0
15. Cancer	Yes = 1, no = 0
16.Reproductive system disorders	Yes = 1, no = 0
17.Other diseases	Yes = 1, no = 0
Geriatric symptoms	
18.Urinary incontinence	Yes = 1, no = 0
19. Faecal incontinence	Yes = 1, no = 0
20.Fall history	Yes = 1, no = 0
21.Visual impairment	Normal = 0, mildly impaired = 0.25, moderately impaired = 0.5, severely impaired = 0.75, almost blind = 1
22.Hearing impairment	Normal = 0, mildly impaired = 0.5, severely impaired = 1
Health status and emotion	
23. How do you feel about your health status?	Excellent = 0, good = 0.25, fair = 0.5, not good = 0.75, poor = 1
24. Do you currently need someone to take care of you in your daily life?	Yes = 1,no = 0
25.Do you feel lonely༟	Often = 1, sometimes = 0.5, never = 0
26. How happy do you feel?	Very happy = 0, happy = 0.25, moderately happy = 0.5, unhappy = 0.75, very unhappy = 1
Use of assistive devices	
27.Hearing aids	Yes = 1, no = 0
28.Dentures	Yes = 1, no = 0
29.Crutches	Yes = 1, no = 0
30.Wheelchair	Yes = 1, no = 0
31.Adult diapers/pads	Yes = 1, no = 0

*Abbreviations*: *ADL* Activity of daily living
